# Radiation-induced alterations in multi-layered, in-vitro skin models detected by optical coherence tomography and histological methods

**DOI:** 10.1371/journal.pone.0281662

**Published:** 2023-03-02

**Authors:** Luisa Bromberger, Bettina Heise, Karoline Felbermayer, Elisabeth Leiss-Holzinger, Katarina Ilicic, Thomas Ernst Schmid, Alexandra Bergmayr, Tanja Etzelstorfer, Hans Geinitz

**Affiliations:** 1 Department of Radiation Oncology, Ordensklinikum Linz Barmherzige Schwestern (BHS), Linz, Austria; 2 Institute for Mathematical Methods in Medicine and Data Based Modelling, Johannes Kepler University (JKU), Linz, Austria; 3 Research Center for Non-Destructive Testing (RECENDT)-GmbH, Linz, Austria; 4 Department of Radiation Oncology, Klinikum rechts der Isar (MRI), TUM München, München, Germany; 5 Department of Pathology, Ordensklinikum Linz Barmherzige Schwestern (BHS), Linz, Austria; Affiliated Hospital of Jiangsu University, CHINA

## Abstract

**Background:**

Inflammatory skin reactions and skin alterations are still a potential side effect in radiation therapy (RT), which also need attention for patients’ health care.

**Method:**

In a pre-clinical study we consider alterations in irradiated in-vitro skin models of epidermal and dermal layers. Typical dose regimes in radiation therapy are applied for irradiation. For non-invasive imaging and characterization optical coherence tomography (OCT) is used. Histological staining method is additionally applied for comparison and discussion.

**Results:**

Structural features, such as keratinization, modifications in epidermal cell layer thickness and disorder in the layering—as indications for reactions to ionizing radiation and aging—could be observed by means of OCT and confirmed by histology. We were able to recognize known RT induced changes such as hyper-keratosis, acantholysis, and epidermal hyperplasia as well as disruption and/or demarcation of the dermo-epidermal junction.

**Conclusion:**

The results may pave the way for OCT to be considered as a possible adjunctive tool to detect and monitor early skin inflammation and side effects of radiotherapy, thus supporting patient healthcare in the future.

## Introduction

At least half of all cancer patients receive ionizing radiation as part of their treatment [1–3]. Despite the effective tumour control of radiation therapy, the risk of acute and late side effects remains a challenge [4, 5]. Skin alterations and in particular, skin damages are an undesirable side effect of radiotherapy. Radiation dermatitis can occur as a side effect of ionizing radiation [6–8]. Although the frequency and severity of skin toxicity is continuously decreasing because of improved radiation therapy settings and planning schemes, it remains a problem in certain patient groups. This is especially the case for patients receiving radio- or radiochemotherapy for head and neck cancers [9–11].

The cells of the basal layer of the epidermis of the skin are rapidly dividing and therefore are prone to cell death upon radiation therapy [12]. In addition, an inflammatory reaction evolves, resulting in an erythematous reaction [13]. Inflammation is the first acute response of the skin. These lesions may occur depending on tumour location, radiation technique, total dose and volume, fraction, concurrent systemic therapy, concomitant diseases, as well as individual (X-ray) radiation sensitivity and genetic predisposition [14–16]. Typical skin reactions after radiotherapy include inflammatory symptoms such as swelling, redness, pain, burning and itching. However, pigmentary changes, loss of hair follicle stem cells and, in rare cases, ulceration and fibrosis may also be observed [17–19]. Overall, skin reactions can be classified as acute or chronic and occur depending on intrinsic and extrinsic risk factors [17, 18]. Acute skin reactions typically occur within one to four weeks after the start of radiotherapy (RT) and may persist during treatment [14], see Table 1, where acute human skin reactions are listed.

**Table 1:** Human skin reactions.

Observed acute skin reaction	Radiation dose [Gy]	Time
Transient erythema	2	hours
Faint erythema and epilation	6–10	7–10 days
Defined erythema and hyper-pigmentation	12–20	2–3 weeks
Dry desquamation	20–25	2–3 weeks
Moist desquamation	30–40	> 4 weeks
Ulceration	>40	> 6 weeks

Dose-dependent cutaneous acute human skin reactions after radiation (normo-fractionated, 2Gy per fraction) [14], described further in [18, 20].

We will focus here on morphological features described for acute radiation dermatitis. Despite the huge technological advances in radiation oncology in recent years, higher grade radiation dermatitis might interfere with the patient‘s quality of life and sometimes limits the applied cumulative radiation dose [19].

Optical Coherence Tomography (OCT), which has been introduced originally in ophthalmology in the 1990’s [21, 22], is a non-invasive, low coherent imaging technique. It operates typically in near infrared spectral range and with light of moderate intensity. OCT has found its entrance as medical diagnosis supporting tool, which can enable “optical biopsies” by imaging [23]. There is a wide range of reported applications of OCT [24] in dermatology, used e.g. for characterization of skin photodamage/photosensitivity [25, 26] or wound healing processes [27, 28], and in cutaneous oncology [29–32]. OCT might also be applied for the detection and surveillance of cutaneous side effects of medical procedures [33–36]. Conventional OCT is increasingly supplemented by angiographic OCT modalities in order to further characterize vascular changes [37–39]. We anticipate that OCT could also be used in the future as a promising diagnostic and/or predictive tool for characterizing individual radiation-induced human skin reactions due to radiation therapy. However, before OCT can be introduced in a clinical study as a supportive tool for grading and predicting individual human radiation sensitivity of patients, preclinical studies need to be taken along the way to test OCT in radiation therapy-like application schemes, realized at animal models [40] or at in-vitro skin equivalents. The use of skin-equivalent tissue models and their imaging by OCT has already been demonstrated, for example, in the context of wound healing [41] or for monitoring the effects of laser thermal therapy [33] or tissue engineering [42, 43].

Our investigations were carried out in an in-vitro skin equivalent tissue model consisting of human epidermal and dermal cell layers (briefly called ‘in-vitro skin model’ in the following) in order to account for the preserved three-dimensional geometric arrangement and communication of cells present in tissues. The aim of this work is to identify radiation-induced reactions and responses in the in-vitro skin models and to discuss the ability of OCT to visualize and characterize these alterations.

Since OCT is a relatively new method in the wide context of radio-oncology [44–50], diagnostic algorithms and procedures - similar to other imaging modalities—need to be established to make the examination as objective as possible. Appearing features and algorithms need to be constantly re-evaluated during this process. Therefore, also a histological staining method is used in comparison to OCT imaging. This procedure might support a prospective clinical use of OCT for monitoring radiotherapy patients to radiation sensitivity and toxicity.

## Materials and methods

Since in this pre-clinical study we aim to visualise radiation-induced lesions, alterations, and suspicious features in the multicellular model that are predictive of the development of radiation dermatitis, we used both non-invasive OCT imaging and a comparative histological method, although the latter requires biopsy/sectioning and staining.

### Specimens

EpiDerm Full Thickness 400 (EFT-400) skin equivalent tissue models from MatTek Cooperation [51] (Lot. 021918GSB) were used for all radiation exposure experiments. The EFT-400 system consists of normal human epidermal keratinocytes (NHEK) and normal human dermal fibroblasts (NHDF), however without vascular structures. They form a multi-layered, differentiated specimen consisting of basal, spinous, granular and cornified cell layers, partly resembling the normal human epidermal as well as a dermal layer arrangement. The missing of microvascular structures represents a limitation of this in-vitro model. However, the model is well-established for testing to radiation toxicity and alterations. Furthermore, applying skin models for radiotherapy experiments is also an option for avoiding/reducing the use of animal models [52].

**Fig 1:**
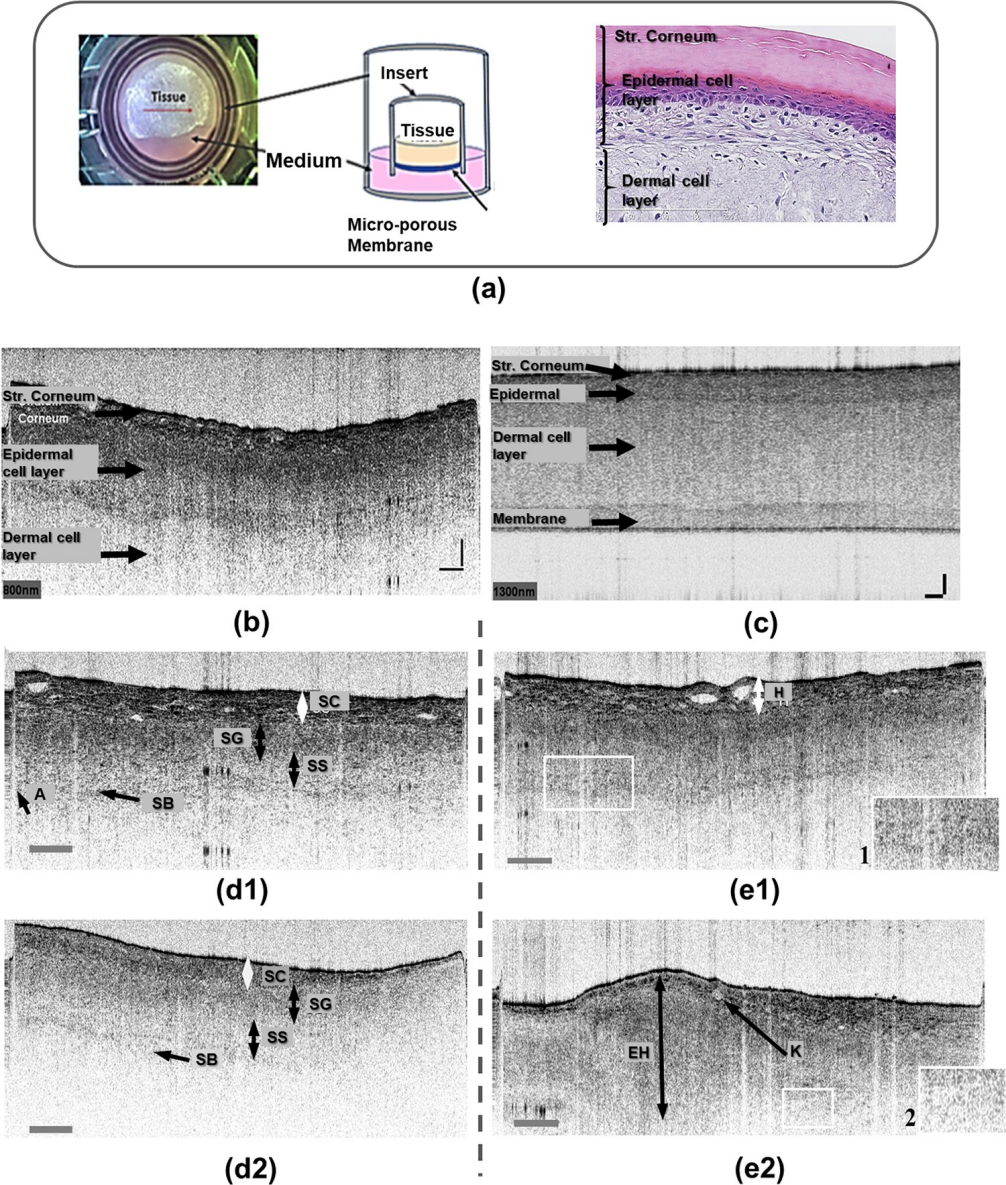
OCT imaging-based insights. (a) The EpiDerm Full Thickness in-vitro skin model, under OCT imaging stage and tissue culture scheme according to MatTek [51], and histological section scheme consisting of epidermal keratinocytes layers (basal, spinous, granular, and cornified cell layers) and dermal fibroblasts layer; Cross-sectional OCT scans for: (b): 800 nm and (c): 1300 nm central wavelength, exemplified at the in-vitro skin model. Note the competing properties of higher resolution (b) vs. deeper penetration (c), due to different spectral probing ranges; (d) Cross-sectional OCT scans of *non-irradiated* in-vitro skin model samples at 2 days after exposure; (e) OCT scans, illustrating morphological alterations at *irradiated* in-vitro skin models. Indicated features: (d) cornified (SC); granular (SG); spinous (SS); basal (SB)—cell layers; boundary artefact (A); (e) hyperkeratosis (H), epidermal hyperplasia (EH), keratosis (K). Enlarged details are shown in the insets: 1) cellular layer interface; 2) scattering structures; for more details see also S1 File. Scalebars (h/v): (b) and (c) resp.: 150/50 and 100/100 μm resp. f = 30 mm. Central wavelength OCT: (d), (e) 800 nm. All OCT scans are depicted in inverted brightness representation, i.e. hyper-reflective surface [31, 57] is expressed by a dark contour.

The medium (Dulbecco’s Modified Eagle’s Medium (DMEM)) containing epidermal growth factor, insulin, hydrocortisone and other proprietary stimulators of epidermal differentiation, gentamicin 5 μg/ml, amphotericin B 0.25 μg/ml, phenol red and lipid precursors) and the 6-well plates used were supplied with the EFT-400 kit, see also Fig 1(A). After delivery of the tissues, new 6-well plates were prepared by adding 2.5 ml of the medium to each well using sterile technique in a tissue culture bonnet. Using sterile forceps, the gauze cover was removed from the insert again and each EFT-400 tissue was inserted into a well. No agarose adhered to the insert. The medium was completely in contact with the underside of the membrane of the tissue insert.

The in-vitro skin models were cultured in a humidified incubator at 37°C and 5% CO2 and transferred usually every 24 hours. The medium was stored in a refrigerator at temperatures between 2 and 8°C and pre-warmed in a water bath before use.

Immuno-histochemical analyses were performed according to the standard protocols to detect characteristic changes that are typical in natural skin when exposed to radiation and occur regularly in the investigated model. However, for the in-vitro model some specific proliferation and ageing patterns [53] have to be considered.

### Radiation scheme

The skin models (in total 12 specimens; 6 each for BHS, Linz and for MRI, Munich) were exposed to radiation 5 days after delivery. The specimens were non-irradiated (0 Gy, samples for comparison) or irradiated with 2 Gy (typical single dose in fractionated radiation schemes) or 10 Gy (typical dose applied in stereotactic radiation schemes), 2 samples each. The exposure was realized with 6 MeV electrons of a linear particle accelerator (Clinac iX, Varian Medical Systems). To achieve a homogeneous dose distribution, a field of 20 x 20 cm^2^ was applied. In addition, 1.5 cm thick water equivalent plates were used to ensure efficient dose transfer to the samples. After irradiation, the samples were transferred to new 6-well plates with fresh medium.

The samples were nourished and stored in the incubator for 48 hours and 96 hours. Then the OCT measurements were performed in case of the day 2 and day 4 investigations.

During the time course of the investigation the model tissues are also undergoing aging processes, which can affect the cornified cell layer [54] additional to the irradiation. To discriminate best between both simultaneously occurring processes and changes, we perform OCT imaging and histology for non-irradiated tissue models (0 Gy, control samples) in the same temporal course, which will allow at least a qualitative description of possible alteration and responses in the investigated models.

### OCT imaging

A customized OCT system (Fourier Domain OCT operating at 800 nm central wavelength, axial resolution in air 2 μm) and a commercial OCT system (Thorlabs, Telesto OCT system, operating at 1300 nm central wavelength, axial resolution in air 7 μm) have been applied for OCT imaging and examination. These two wavelength ranges have shown promise for the examination of the multi-layered, in-vitro model in terms of resolution and imaging penetration depth, respectively. Cross-sectional images, so-called B-scans, have been recorded at the irradiated (and comparatively at the non-irradiated) in-vitro models.

### Histology

The OCT-imaged in-vitro skin models have been analysed subsequently by histological examinations (at BHS, Linz). Therefore, the inserts were removed from the skin model. The tissues were then placed in formaldehyde and fixed for 16 hours. They were stored in non-sterile phosphate buffered saline (PBS) for up to 72 hours until further processing. The samples were embedded in paraffin wax and cut with a layer thickness of 5 μm.

HE stains as well as immunohistochemistry were used to examine the features and alterations in the in-vitro skin model with the aim of establishing criteria to illustrate responses to radiation and arising of early radiation dermatitis.

The HE staining was performed using standard protocols. Cell proliferation staining was performed with the primary Antibody Anti-Ki67 SP6 Rabbit Abcam, using a 1:100 dilution. The samples were pre-treated by incubation with ER1 buffer (citrate buffer) for 20 min. Then they were treated with Peroxide Block (5 min, room temperature (rt.)), Marker Reagent (15 min, rt.), Polymer Reagent (8 min, rt.), Mixed DAB Refine (10 min, rt.), and counter stained with Hematoxylin (5 min, rt.) (all from Leica Microsystems). The Ki67 protein is expressed in G1, S, G2 and M phase of the cell cycle, however, it is not detectable in G0 and early G1 phase [55]. In pathology, the antibody is used to distinguish benign from malignant lesions and for tumor grading. Often, higher levels of Ki67 expression correlate with a poor prognosis. Ki67 labeling index is a widely used marker to determine the growth fraction of a given population [56].

### Statistical analysis

For the estimation of the layer thickness, the distances between the visible cell layers were repeatedly measured manually with the software tool ImageJ. The GraphPad Prism 8 Software was used to calculate the standard error of the means, to perform the descriptive statistical calculations and a one-way analysis of variance (ANOVA). The ANOVA test is used to compare the means of at least two different groups. The alternative hypothesis is accepted if the means of at least two groups are statistically different from each other. The level of significance was set to 5%.

## Results

### Morphological findings at in-vitro skin model by OCT imaging

In comparison to OCT imaging operating at 800 nm central wavelength, deeper skin layers can better be recognized by OCT at 1300 nm central wavelength, as illustrated in Fig 1(B) and 1(C). For visualization of the full thickness of the in-vitro model, i.e. also the underlying membrane, a probing at 1300 nm is more favourable. However, the axial and lateral resolution at 1300 nm is worse in comparison to the 800 nm OCT, which leads to reduction of detailed information on particular cellular layers alterations. Furthermore, the water absorption at 1300 nm is stronger than those at 800 nm central wavelength. Hence, a suitable choice of spectral probing range in OCT must be taken, which in our case has been mainly a use of OCT at 800 nm.

For our investigations, we have to carefully distinguish between conspicuous features arising in the epidermal and dermal layers of the model and morphological alterations due to ageing and those due to radiation treatment. Therefore, we also considered the non-irradiated samples in parallel to the irradiated samples under the same longitudinal course.

For non-irradiated samples typical morphological features are evident in the epidermal cell layer, in particular the stratification of the epidermal layer, which was investigated here using OCT imaging at 800 nm, with a focal distance of 30 mm and especially of 19 mm. The arrangement of the layers was determined here visually by comparing the changes in scattering features expressed by different random speckle-like appearances. The cross-sectional OCT scans in Fig 1(D) show the multi-layer appearance (cornified, granular, spinous, basal cell layers) of the non-irradiated skin model in this imaging technique. A clear transition from the epidermal cell layer to the dermal cell layer, here below the basal cell layer, is not always visible. However, in most OCT images of non-irradiated samples, it is possible to identify the dermal-epidermal junction layer. Additional potential alterations, found by 800 nm OCT for irradiated samples, point to features like hyper-keratosis, acantholysis, and epidermal hyperplasia, as shown in Fig 1(E).

### Structural findings at in-vitro skin model by histology

**Fig 2:**
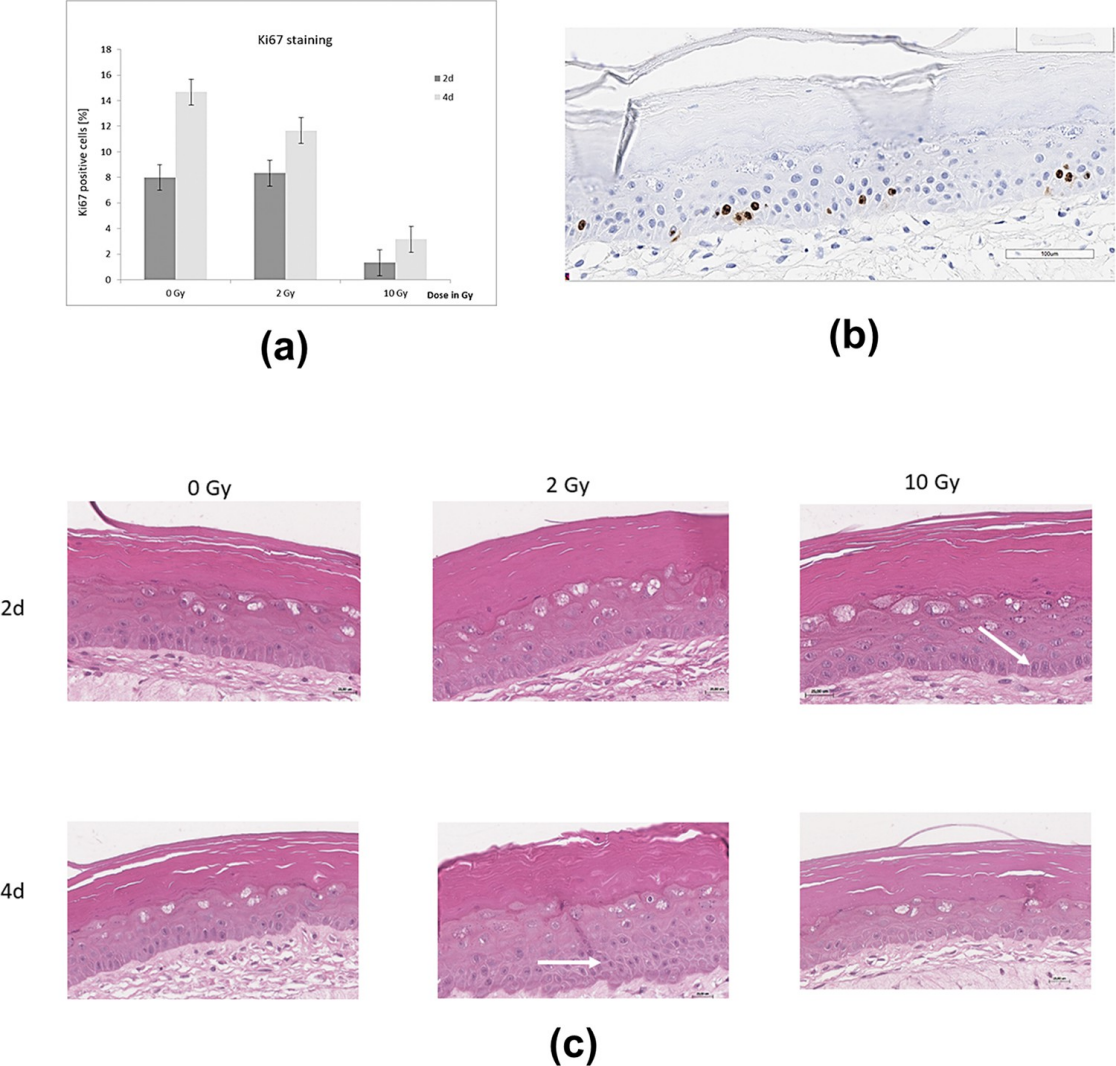
Histological insights. (a) Proliferation diagram by (Ki67) staining after irradiation (2d = 2 days; 4d = 4 days); (b) Histological examination of Ki67 immunostained sections of the epidermal layer of the in-vitro skin model showing positive (brown cells) or negative (blue cells) in the epidermal layer; (c) HE stained histological sections, illustrating morphological alterations in the *non-irradiated* skin models (0 Gy, control samples) and in the *irradiated* in-vitro skin models, at day 2 and day 4 after exposure, irradiated by 2 Gy, and 10 Gy, (H&E staining). At day 2 and still more at day 4 after exposure a discrete (reactive) thickening of basal epidermal cell layer has been seen, (indicated with white arrows). Vacuolation of cytoplasm is most prominent at day 2 after exposure. At day 2 and day 4 an increasing hyperkeratosis is noted, caused probably due to both effects: irradiation and aging.

In addition to the morphological findings we also analyzed structural alterations by immuno-histochemistry. Ki67 is a widely used marker to determine the growth fraction of a given population. In pathology, the antibody is also used to distinguish benign from malignant lesions and for tumor grading. Here, higher levels of Ki67 expression correlate with a poor prognosis. Proliferation also often increases in normal tissue due to regenerative and reparative processes after tissue damage. In the present studies, we used a full-thickness skin equivalent to further characterize the effects of radiation on keratinocyte proliferation. Radiation exposure of the skin can cause erythema, edema, or desquamation within several days. Fig 2(A) shows the results of the Ki67 staining. Ki67 levels were generally higher four days after irradiation than two days after irradiation. A significant decrease of Ki67 was seen after 10 Gy irradiation on day 2 and after 2 Gy and 10 Gy irradiation after day 4. The histological examination of the model with prominent epidermal layers is depicted in Fig 2(B). Histological examination of in-vitro skin model, immunostained for Ki67 showing positive (brown cells) or negative (blue cells) in the epidermal layer.

Identical irradiation experiments and following HE staining analysis have been performed at MRI, Munich for comparison. The results are shown in Fig 2(C). Further morphological alterations, such as intracellular edema and hyperkeratosis, as typical features of the irradiated samples are also shown in S1 File.

### Statistical OCT image data analysis for layer thickness estimation

The statistical OCT image data analysis was performed at 6 skin model samples at BHS, Linz. Here, the interest has been directed to overall epidermal and cornified layer thickness values, observed with the OCT system at 800 nm central wavelength and 19 mm and 30 mm focus length resp. A data set and details for the underlaying statistical analysis can be found in S1 File as well as a comparison between OCT and histology-based results.

### OCT measurements (central wavelength λ = 800 nm, f = 19 mm)

*Epidermal layer thickness*. *Two days* after irradiation, the *overall epidermal layer thickness* in the in-vitro skin model increased when the samples were exposed to higher radiation dose compared to non-irradiated ones. The *average epidermal layer thickness* yields:

at 0 Gy: 157 μm, at 2 Gy: 180 μm, and at 10 Gy: 170 μm, with a p-value<0.0001.

**Fig 3:**
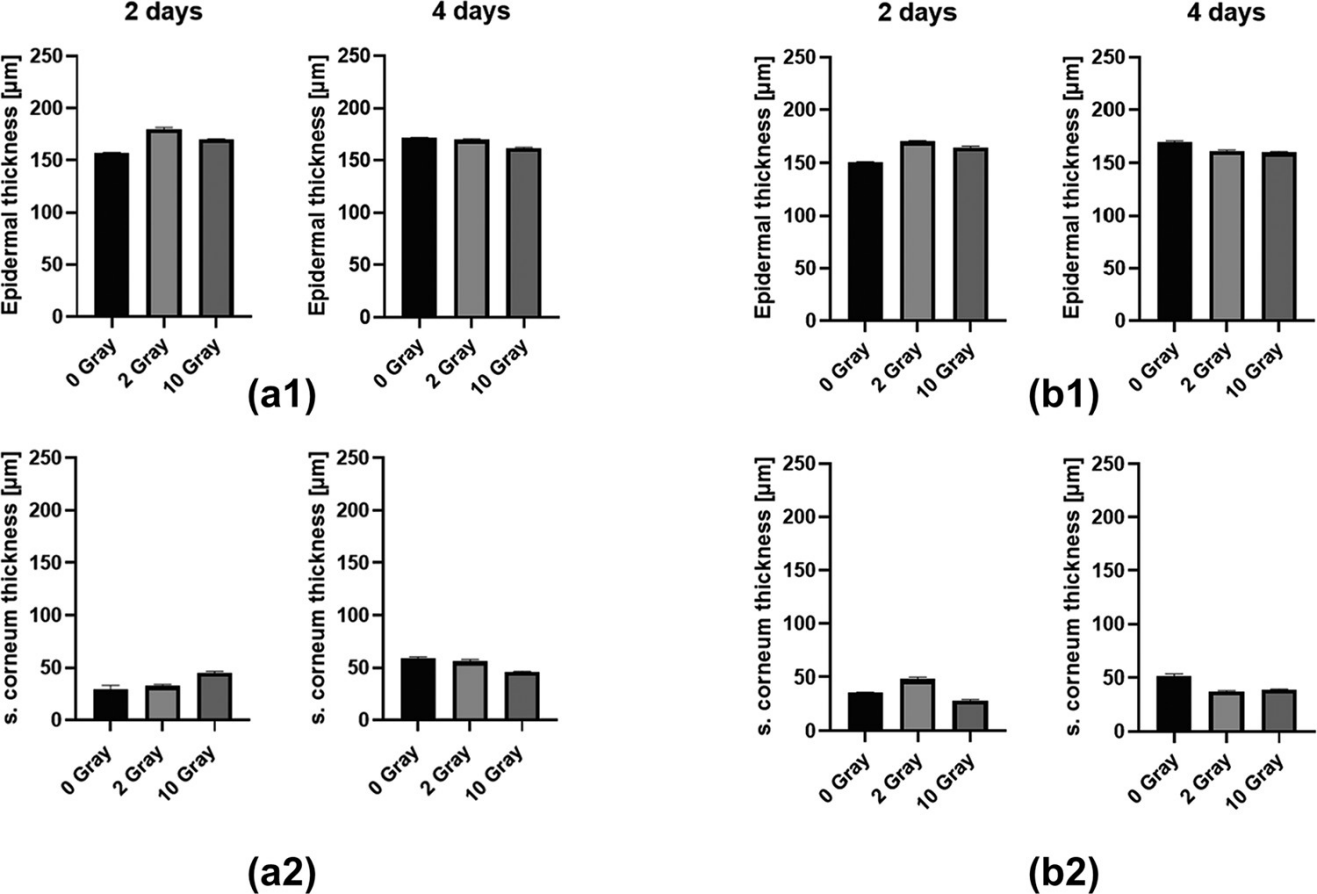
Graphical representation of layer thickness estimation, for (a1, b1): epidermal cell layer thickness, (a2, b2): cornified cell layer thickness, each at day 2 and day 4, without / after irradiation, obtained in OCT measurements using different focal length in optics: (a) λ = 800 nm, f = 19 mm; (b) λ = 800 nm, f = 30 mm.

*Four days after* irradiation, however, the *overall epidermal layer thickness* decreased when the samples were exposed to higher radiation dose. The *average epidermal layer thickness* yields: At 0 Gy: 171 μm, at 2 Gy: 170 μm, and at 10 Gy: 162 μm, with a p-value<0.0001, as depicted in Fig 3(a1).

*Cornified cell layer thickness*. *Two days* after irradiation, the thickness of the *cornified cell layer* first increased with increasing radiation dose: at 0 Gy: 30 μm, at 2 Gy: 33 μm, at 10 Gy: 45 μm, with a p-value = 0.0002.

*Four days* after irradiation, a decrease in thickness of the *cornified cell layer* with increasing dose was observed: at 0 Gy: 59 μm, at 2 Gy: 56 μm, at 10 Gy: 46 μm, with a p<0.0001, as depicted in Fig 3(a2). Over time (day 4 vs. 2) the thickness of *cornified cell layer* of non-irradiated samples increased (additionally by aging).

### OCT measurements (central wavelength λ = 800 nm, f = 30 mm)

*Epidermal layer thickness*. *Two days* after irradiation, the *overall epidermal layer thickness* again increased with higher radiation doses compared to non-irradiated sample. The *average epidermal layer thickness* yields: At 0 Gy: 151 μm, at 2 Gy: 170 μm, and at 10 Gy: 165 μm, with a p-value<0.0001.

*Four days* after irradiation, the *overall epidermal layer thickness* decreased when the samples were exposed to higher radiation dose. The *average epidermal layer* thickness yields: At 0 Gy: 169 μm, at 2 Gy: 162 μm, at 10 Gy: 161 μm, with a p-value<0.0001, as shown in Fig 3(b1).

*Cornified cell layer thickness*. *Two days* after irradiation, an increase of thickness of the *cornified cell layer* was observed for exposure of 2 Gy and a decrease for exposure of 10 Gy compared to the non-irradiated samples: 0 Gy: 36 μm, 2 Gy: 48 μm, 10 Gy: 29 μm, with a p-value<0.0001.

*Four days* after irradiation, the thickness of the *cornified cell layer* decreased compared to non-irradiated samples: 0 Gy: 52 μm, 2 Gy: 38 μm, 10 Gy: 39 μm, with a p-value<0.0001, as shown in Fig 3(b2). Over time (day 4 vs. 2) the thickness of *cornified cell layer* of non-irradiated samples increased again (by aging).

## Discussion

### Expected patterns

According to literature the following features and skin alterations are potentially expected:

- Atypical signal patterns, e.g. atypical honeycomb patterns [58];
- Rapid attenuation of light, lower penetration depths (due to scattering) [31, 58]
- Disruption of the normal layered skin [31, 58], e.g., disruption of the stratum corneum, defined by an irregular entrance signal [58], architectural disarray in the stratum granulosum/ stratum spinosum [58];
- Thickening or disarray of the epidermis [57];
- White streaks in the upper epidermis corresponding to hyperkeratotic areas [57];
- Disruption and/or demarcation of the dermal epidermal junction [6];
- Cellular alternations including pleomorphism of keratinocytes, dyskeratosis and acantholysis as well as cellular and/or nuclear polymorphism (in the stratum granulosum/ stratum spinosum [31, 58];
- The presence of inflammatory cells in the dermis, necrotic/cystic cavities [58];
- Adenexal involvement [31];

### OCT based insights

In the recorded OCT images several of these features could be confirmed for the tested multi-layered, in-vitro skin model. Especially such patterns indicating irregular/disarrayed epidermal layers and deformed dermo-epidermal junction have been found. Morphological changes verified by OCT imaging included hyperkeratosis, acantholysis, epidermal hyperplasia / epidermal thickening, epidermal fissures, and dermo-epidermal junction disordering within layered composition. The thickening of cornified cell layer might be caused both by in-vitro model aging and irradiation. It would need further investigation to discriminate the statistical effects in a quantitative way.

Other, in the RT literature reported prominent skin changes [46, 48], such as alterations and reduction of capillary vessels could not be verified in this study, since the multi-layer in-vitro model is lacking microvascular structures and certain cell types. Therefore, we anticipated to only find reactions that involve fibroblasts or keratinocytes as well as the layering of the skin. Due to the relatively short cultivation period, we did not expect to find chronic reactions that typically require several weeks or months to develop.

### Histology based insights

Compared morphological changes, as also described in literature [18, 19], and verified in the HE stains included intracellular edema, para/hyperkeratosis as well as epidermal hyperplasia, epidermal duplication, keratotic tongues and central keratosis (observed in tests at BHS, Linz). Vacuolation is also described as a typical feature [59] and has been observed by histological examination (both due to irradiation and aging in the temporal course of this study).

Additionally, we found suprabasale fissuring, intradermal fissuring both in OCT and HE staining which might also be caused by skin model sectioning. We did not see signs of hydropic degeneration of the basal layer or edematous dermis as described by [60].

Unlike normal skin, the investigated model does not contain any stem cells [51, 53]. This means, that both keratinocytes as well as fibroblasts will only undergo mitosis over a certain period of time, before losing this ability. Also, depending on the individual cell, some cells will be able to undergo mitosis more often than other cells. Overall, this could result in one cell proliferating two or three times more often while an adjacent cell in the same layer might stop. This selective proliferation could lead to the effect of one part of a layer duplicating, while other parts are thinning. Results of this phenomenon could be the development of keratotic tongues or the duplication of epidermis. This is supported by the findings of parts of layers that appear older than adjacent parts. However, these suggestions need to be supported by more experiments.

Keratinization, which is also observed in human skin conditions [61], could be another parameter when observing radiation dermatitis in patients additionally to the change of stratum corneum thickness. Keratinization often changes the scattering coefficient. This, of course, can only be observed in OCT imaging. Both, characterizing the scattering coefficient and estimating attenuation coefficient by the intensity decrease over depth within the skin tissue as well as changes in the epidermal thickening might be promising indicators to predict radiation dermatitis. These quantities could possibly also be measured automatically with OCT in the future.

**Fig 4:**
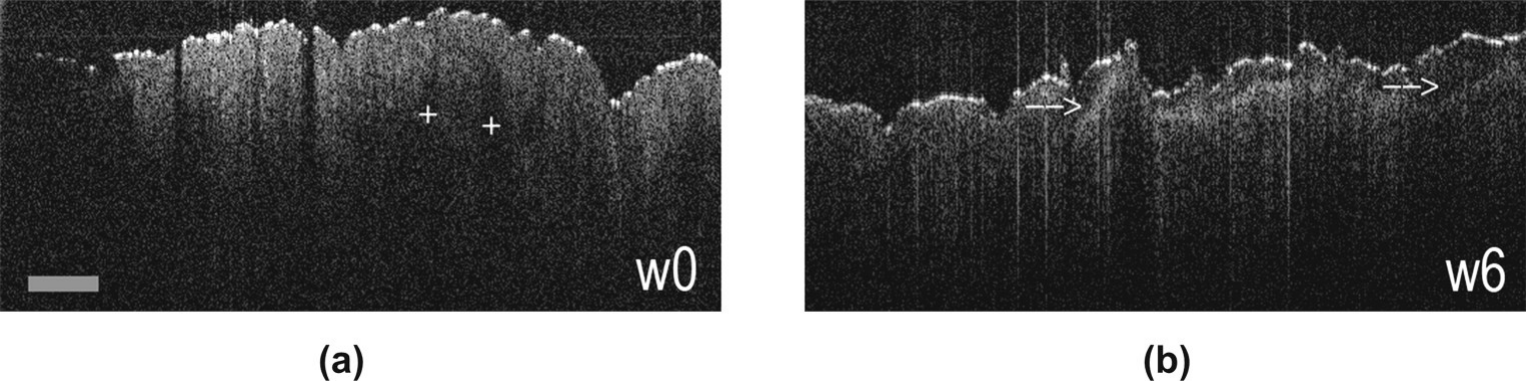
Clinical OCT scans. Cross-sectional OCT skin image recorded at the neck region of a patient undergoing radiation therapy and showing moderate radiation dermatitis symptoms, (a) before beginning (week 0) and (b) at the end (week 6) of RT. Note, before RT the visibility of deeper follicular structures (labelled by +) in a rather homogeneously scattering epidermis, while at week 6 an epidermal /stratum corneum disarray and stronger interfaces (indicated by white arrows) hint on increased keratinization and desquamations. The images are recorded by a commercial OCT system (Lumedica, 800nm central wavelength). Scalebar: 700 μm.

Within a clinical study, which we started after finalizing the herein reported in-vitro experiments, we evaluated the skin changes in patients undergoing radiotherapy for head and neck cancers (https://clinicaltrials.gov/ct2/show/NCT04610645). In that study, the neck skin was imaged longitudinally with a hand-held commercially available OCT scanner. Fig 4 depicts the skin changes observed via OCT in one of the participants rather at the end of RT, showing increased keratinization, desquamation, and epidermal disarray.

### Conclusions

In this paper typical features and findings from OCT imaging were compared to histological findings, exemplified for a multi-layer in-vitro skin model. Indications of hyper-keratosis, acantholysis, and epidermal hyperplasia as well as disruption and/or demarcation of the dermo-epidermal junction could be seen by OCT and have been also confirmed in histology. However, we are also aware the limits using (conventional) OCT—i.e. without an angiographic OCT device—as a diagnostic tool and the use of the in vitro skin model for testing for radiation dermatitis, as microvascular structures, inflammatory cells and hair follicles in particular are missing in the model. These features would be a benefit by using e.g. murine models [40]. The reduction of capillaries and loss of hairs would be suitable morphological features observable in OCT [29, 62] imaging at patients in potential clinical studies.

In general, OCT imaging—as a non-invasive and non-hazardous optical method—can largely be used to visualize morphological changes and alterations in the investigated models. These alterations have been mainly caused by ionizing radiation here, and partly also by aging of specimens. Comparative histological studies have been performed and could partly be correlated with the OCT-based findings.

The described OCT imaging results in combination with further validation of radiation toxicity show the potential to be extended for studies on patients/volunteers in radiation therapy, especially concerning the head and neck regions. We envisage (high-resolution) OCT as a suitable imaging tool to enable an individual monitoring of radiation induced skin lesions and early skin inflammations for patients during radiation therapy. Consequently, such OCT-based monitoring and accompanying of patients in radiation therapy might contribute for a step towards a more personalized concept and individual health care in the future.

## Supporting information

S1 File The file contains additional figures as well the extracted data for the epidermal and cornified layer thickness measurements and the statistical data analysis results thereon. (PDF)
