# Supplementary material for: Radiation-induced alterations in multi-layered, in-vitro skin models detected by optical coherence tomography and histological methods
Source: PLoS One. 2023 Mar 2;18(3):e0281662. doi: 10.1371/journal.pone.0281662 (PMC9980765; doi:10.1371/journal.pone.0281662)
Supplement: S1 File — (PDF) [file pone.0281662.s001.pdf]

Supplementary Information

PONE-D-22-20765R1Radiation-induced alterations in multi-layered, in-vitro skin models detected by Optical Coherence Tomography and Histological MethodsPLOS ONE

In the Supplementary Information file are included:

Figures: S1_Fig 1-4

- S1_Fig 1. OCT imaging (at 1300nm) and histological correlates.
- S1_Fig 2. OCT images of non-irradiated samples
- S1_Fig 3. OCT images of irradiated samples
- S1_Fig 4. Histological sections (1) at irradiated skin models.
- S1_Fig 5. Histological sections (2) at irradiated skin models.
- S1_Fig 6. Thickness of the epidermal and cornified cell layer determined by histological methods.
- S1_Fig 7 Graphical representation of thickness estimation, OCT f=19mm.

Graphical and descriptive analysis data

- S1_Table 1. Results decriptive analysis.
- S1_Table 2. Overview and comparison thickness results.

Data Set

- S1_Table 3. Thickness Measurement OCT, f=16 mm. Data Set

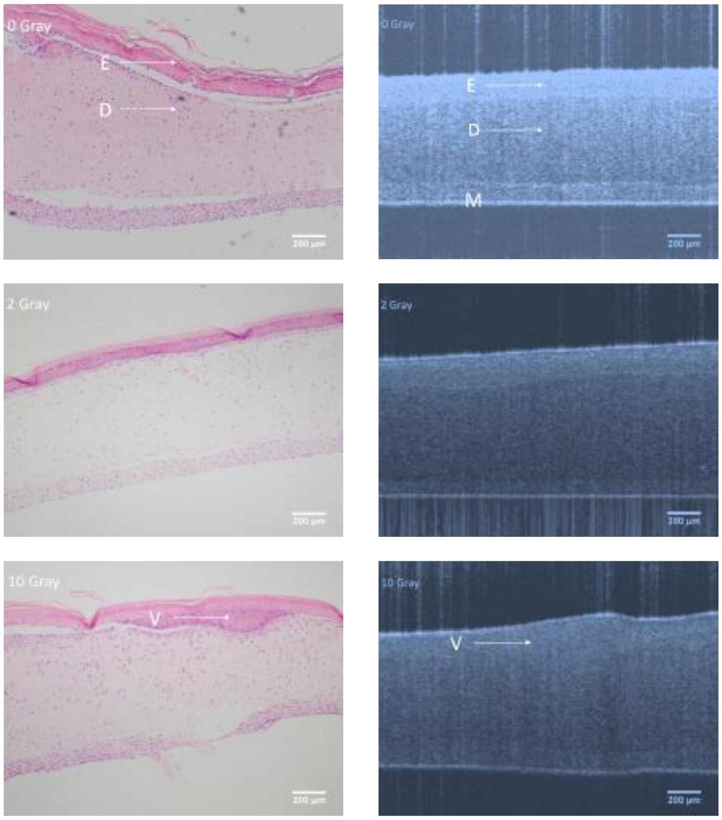

S1_Fig 1. OCT imaging (at 1300nm) and histological correlates. 4 days after radiation; thickening of epithelium (marked as V), in this case epidermal duplication, can be identified clearly histologically, however, cannot be identified as clearly by OCT imaging; epidermis (E); dermis (D); membrane (M); thickening of epithelium (V); typical findings here also include: hyperreflective band (entry signal), a homogenous demarcated darker layer (epidermis), a clear transition to the dermis including a hyperreflective line at the interface between epidermis and dermis could be found.

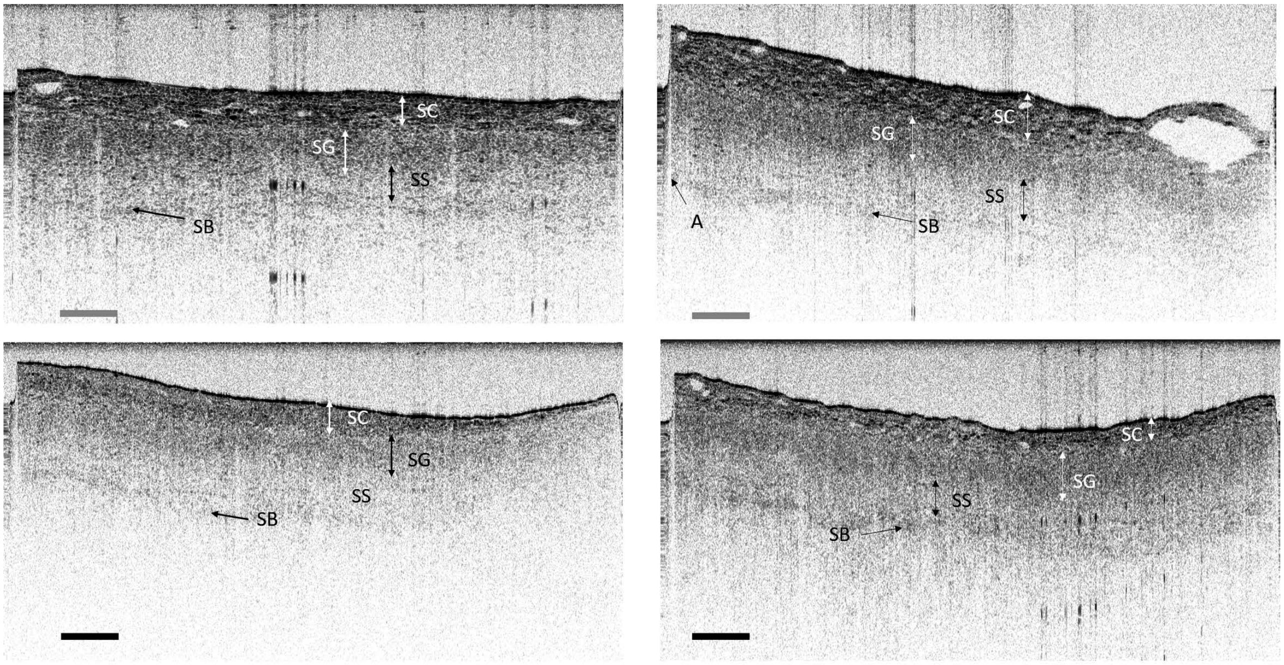

S1_Fig 2. Non-irradiated OCT scans after 2 and 4 days. Cross-sectional OCT scans of non-irradiated in-vitro skin model samples at (left) 2 and (right) 4 days after exposure; Note the thickening of cornified cell layer, also due to model aging. Indicated features: cornified (SC); granular (SG); spinous (SS); basal (SB) - cell layers; boundary artefact (A). (OCT: 800nm central wavelength; focal length: (top) f=19 mm, (bottom) f=30 mm. Scalebar: (top scans): 100 µm; (bottom scans): 300 µm.

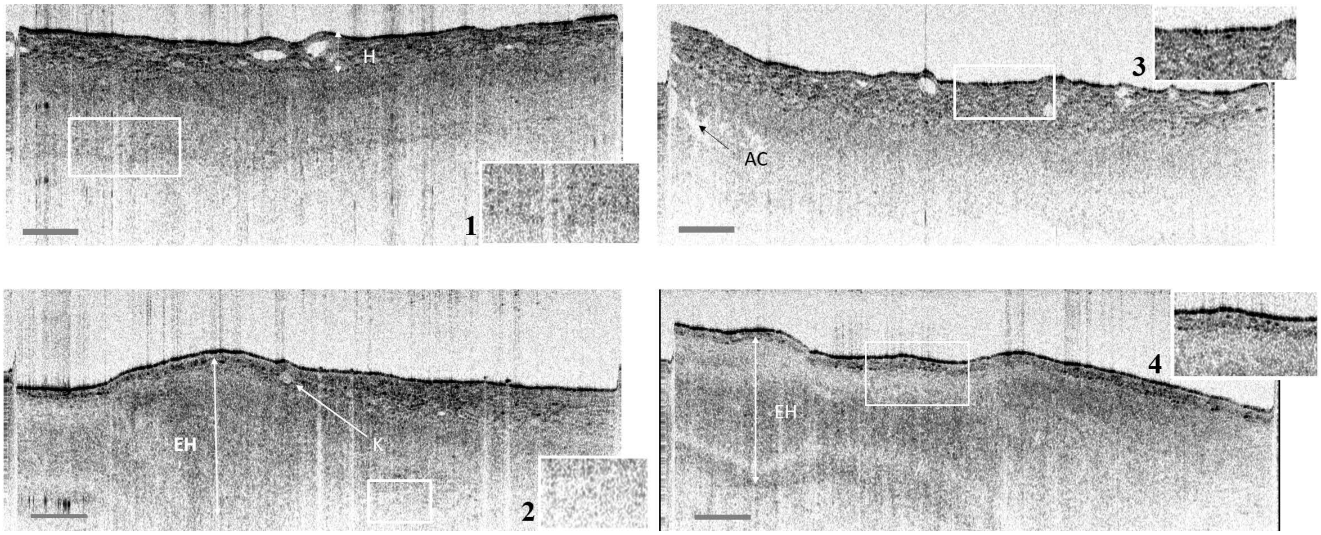

S1_Fig 3. Irradiated OCT scans with alterations. Cross-sectional OCT scans illustrating morphological alterations at irradiated in-vitro skin models, examined by OCT imaging (at 800 nm central wavelength, with (top) f=19 mm, (bottom) f=30 mm focal length). Indicated features: hyperkeratosis (H), acantholysis (AC), epidermal hyperplasia (EH), and (hyper)keratosis (K). Enlarged details are shown in the insets: 1) cellular layer interface; 2) scattering structures; 3) cornified layer; 4) granular details. Scalebar: (top scans): 100 µm; (bottom scans): 300 µm.

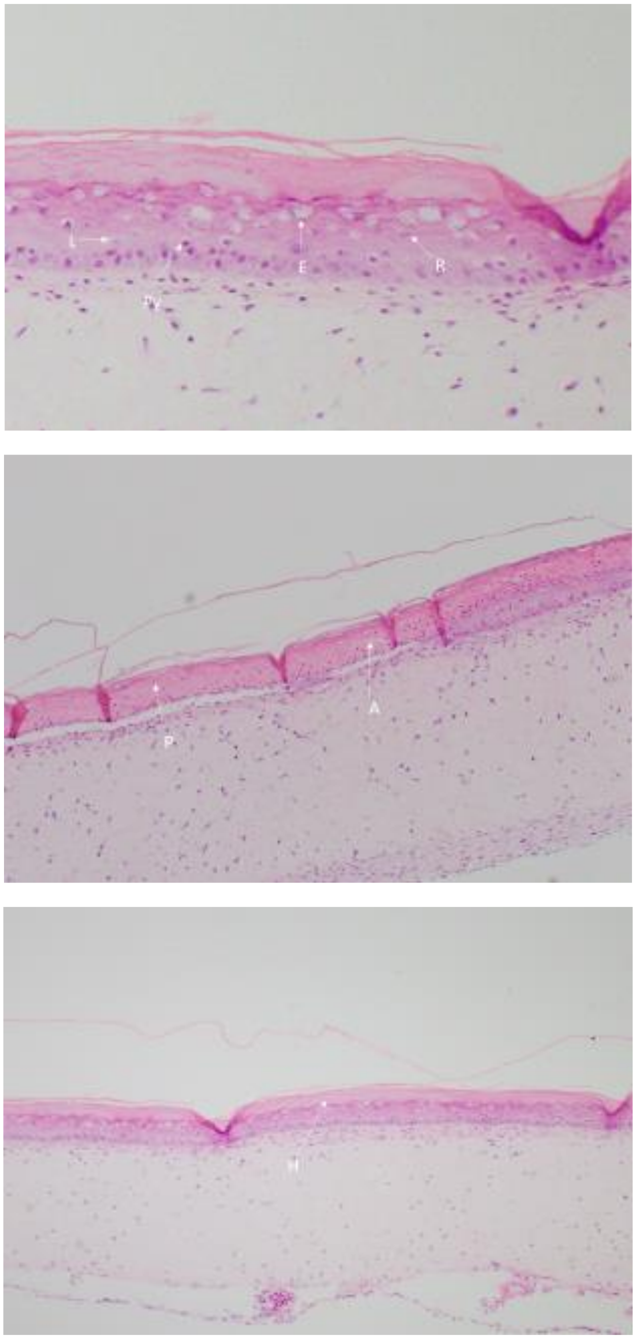

S1_Fig 4. HE-stained histological sections (1) at irradiated skin models. Morphological alterations indicated: karyolysis (L); karyorrhexis (R); pyknosis (Py); intracellular edema (E); acantolysis (A); parakeratosis (P); hyperkeratosis (H).

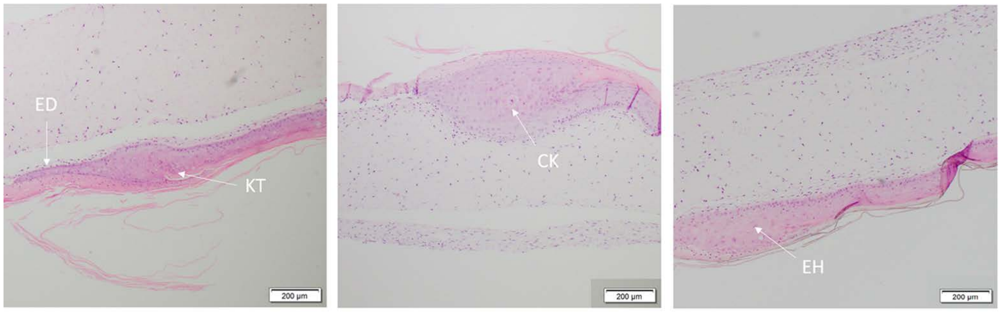

S1_Fig 5. HE stained histological sections (2) at irradiated in-vitro skin models. (2 resp. 10 Gy). Morphological alterations indicated: epidermal duplication (ED), keratotic tongues (KT); central keratosis (CK), intracellular epidermal hyperplasia (EH); Note the depicted alterations additionally due to irradiation might also be affected due to mechanical stress during tissue sectioning and by the influence of aging, respectively.

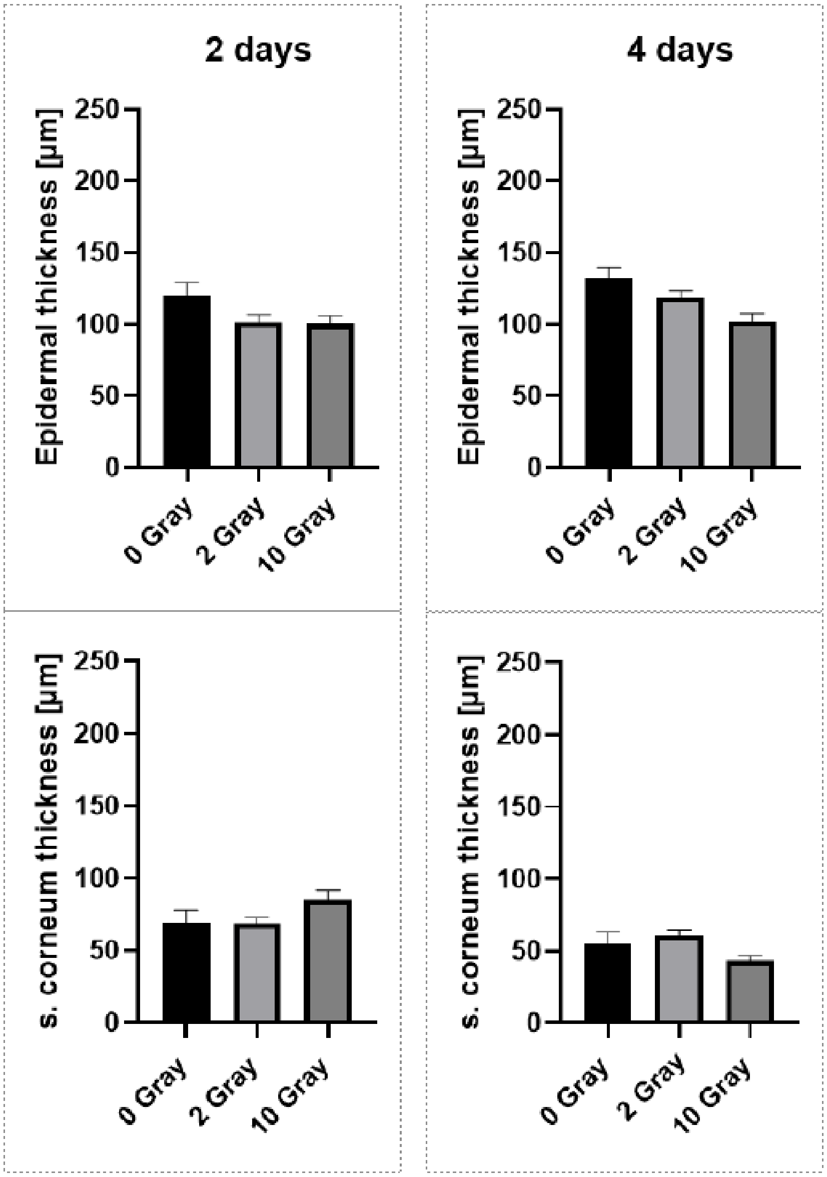

S1_Fig 6. Thickness of the epidermal and cornified cell layer determined by histological methods. After 2 days after exposure, the epidermal layer thickness and the cornidied cell layer did not change significantly with exposure to increasing dose. After 4 days, the epidermal thickness decreased with exposure to increasing dose (p-value 0.0111). The change in the thickness of the cornified cell layer was not significant; epidermial layer after 2 days: 0 Gy: 119µm, 2 Gy: 102µm, 10 Gy: 100µm; p-value=0.1108; after 4 days: 0 Gy: 132µm, 2 Gy: 119µm, 10 Gy: 103µm; p-value=0.0111; cornified cell layer after 2 days: 0 Gy: 69 µm, 2 Gy: 69µm, 10 Gy: 82µm; p-value=0.1185; after 4 days: 0 Gy: 55µm, 2 Gy: 61µm, 10 Gy: 43µm; p-value=0.1065.

Thickness Estimation: Statistical Results

(for f=19mm OCT version)

Graphical Representation

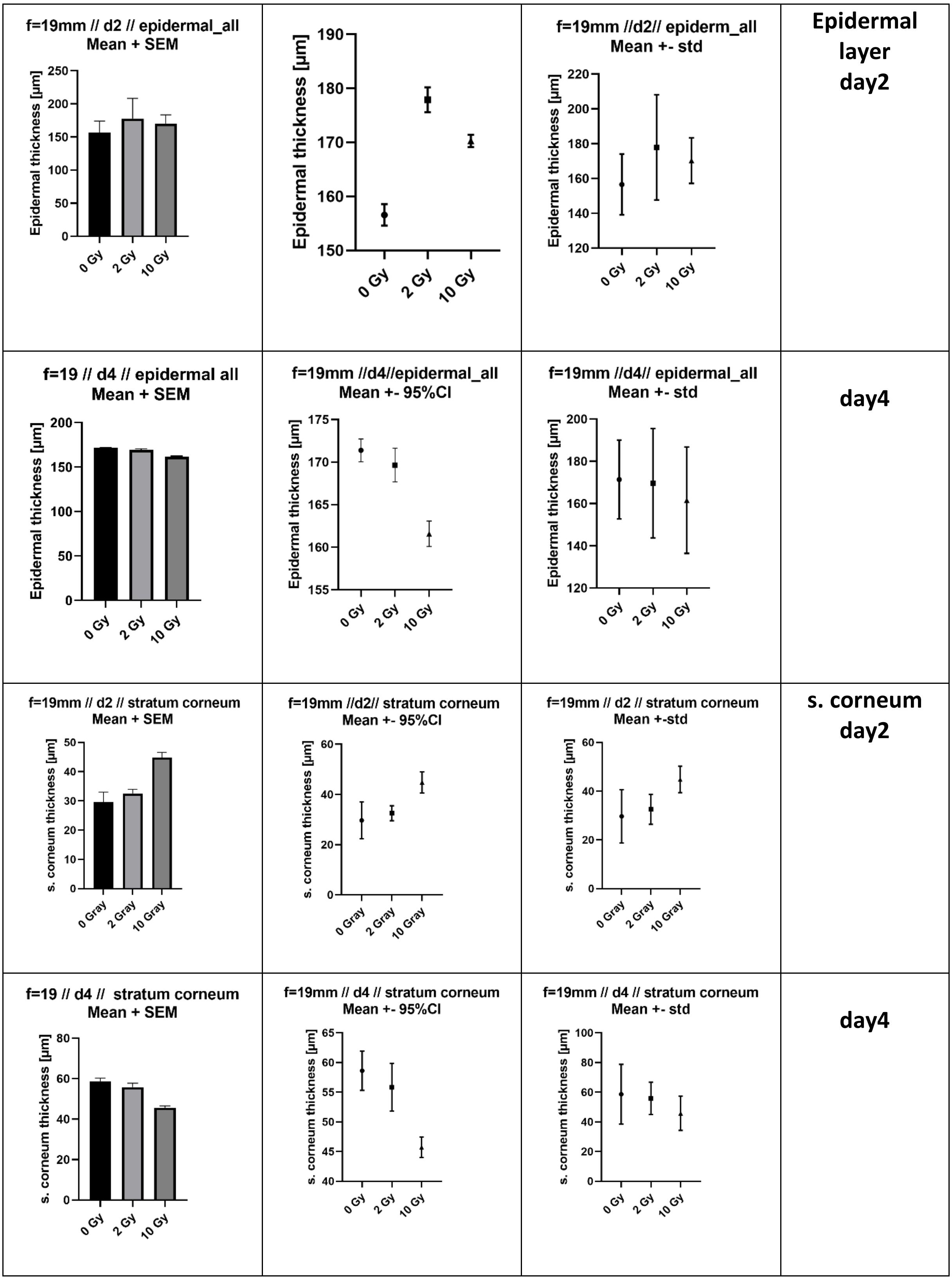

S1_Fig 7. Graphical representation for thickness estimation, OCT f=19mm. Statistics results: epidermal and cornified cell layer thickness estimation, recorded with OCT system at f=19mm focal length; (SEM: Standard error of Mean; CI: Confidence Interval; Std: Standard deviation).

Descriptive Analysis

- performed with Graph Pad Prism Statistics software
- based on OCT measured thickness data - for 0 Gy, 2Gy and 10 Gy - skin model samples

	0 Gy	***2 Gy***	***10 Gy***
Number of val	297	665	503
Minimum	129.2	86.37	136.9
Maximum	249.3	321.6	229.3
Range	120.1	235.2	92.35
Mean	156.6	177.9	170.3
Std. Deviation	17.45	30.24	13.06
Std. Error of M	1.012	1.174	0.5825

Epidermal layer **d2** **f=19mm**

	**0 Gy**	**2 Gy**	**10 Gy**
Number of val	741	666	1091
Minimum	130.8	121.5	89.23
Maximum	240.8	320	300
Range	110	198.5	210.8
Mean	171.4	169.6	161.6
Std. Deviation	18.67	25.92	25.17
Std. Error of M	0.686	1.004	0.7621

Epidermal layer **d4** **f=19mm**

	**0 Gray**	**2 Gray**	**10 Gray**
Number of val	11	19	9
Minimum	16.15	23.08	35.52
Maximum	54.63	44.62	50.79
Range	38.48	21.54	15.27
Mean	29.68	32.56	44.83
Std. Deviation	10.94	6.149	5.462
Std. Error of M	3.298	1.411	1.821

Cornified cell layer **d2** **f=19mm**

0 Gy	2 Gy	10 Gy
147	31	179

Number of val			
Minimum	23.08	30.77	16.06
Maximum	114.9	78.48	69.3
Range	91.8	47.71	53.24
Mean	58.61	55.84	45.72
Std. Deviation	20.16	10.93	11.55
Std. Error of M	1.663	1.963	0.863
**Cornified cell layer**	**d4**		**f=19mm**

S1_Table 1. Results decriptive analysis. Statistical results of thickness estimates for epidermal layer and cornified cell layer, based on OCT B-scan image data (f=19 mm); recorded at day 2 and 4; for non-irradiated, and irradiated skin models (2 Gy and 4 Gy).

		Epidermal layer [μm]			
Time [hrs]	Dose [Gy]	Histology	$\pm SEM[\mu m]$	f = 19 mm	f = 30mm
48	0 Gy	119.3 $\pm$9.8		156.6 $\pm$1.0	150.7 $\pm$0.7
	2 Gy	101.7 $\pm$4.9		180.0 $\pm$1.5	170.3 $\pm$0.4
	10 Gy	100.2 $\pm$5.5		170.2 $\pm$0.5	165.1 $\pm$1.2
P-Value		0.1108		<0.0001	<0.0001
96	0 Gy	132.1 $\pm$7.3		171.4 $\pm$0.7	169.3 $\pm$1.0
	2 Gy	118.6 $\pm$4.7		169.6 $\pm$1.0	161.5 $\pm$1.0
	10 Gy	102.7 $\pm$5.0		161.6 $\pm$0.8	160.1 $\pm$1.1
P-Value		0.0111		<0.0001	<0.0001
		cornified cell layer[μm] $\pm SEM[\mu m]$			
48	0 Gy	69.3 $\pm$8.7		29.7 $\pm$3.3	35.7 $\pm$0.6
	2 Gy	68.7 $\pm$3.7	-	32.6 $\pm$1.4	47.8 $\pm$1.8
	10 Gy	85.3 $\pm$6.2		44.8 $\pm$1.8	28.7 $\pm$0.7
P-Value		0.1185	-	0.0002	<0.0001
96	0 Gy	54.7 $\pm$8.3		58.6 $\pm$1.7	51.6 $\pm$1.7
	2 Gy	60.9 $\pm$3.8	-	55.8 $\pm$2.0	37.5 $\pm$1.0
	10 Gy	43.2 $\pm$3.6	-	45.7 $\pm$0.9	39.1 $\pm$0.7
P-Value		0.1065		<0.0001	<0.0001

S1_Table 2. Overview and comparison thickness results. Epidermal layer and cornified cell layer thickness estimates, obtained by histology and OCT imaging; (Mean +/- SEM. Time = Time from exposure to measurement.) Note the differences in absolute values (between histo/ TU Munich and OCT/ BHS Linz), but the coincidences mostly in trends.

S1_Table 3:
Thickness Measurement : OCT f19mm
Data Set
Measured values [in µm]
Epidermis#
S. Corneum#

- Measured thickness data at multiple B-scans by public image processing tool ImageJ
- Assuming a refractive index of n=1.3 for calculating geometric thickness from optical path length

Thickness Epiderm [µm]; day 2, f19mm

0 Gray	2 Gray	10 Gray
166	144	148
160	157	160
142	142	165
138	191	162
143	245	137
160	142	189
162	160	175
146	174	177
140	197	169
140	251	183
160	151	171
161	160	160
136	163	168
137	180	155
140	240	171
163	138	169
154	161	160
146	169	165
142	195	171
134	245	171
162	140	157
160	165	162
151	165	178
143	179	166
143	249	174
160	174	159
164	212	175
139	260	165
139	289	160
138	276	166
164	217	182
176	271	172
148	289	163
160	311	171
151	265	152
159	197	148
169	180	177
160	177	180
160	246	163
151	286	152
169	182	174
169	189	169
148	212	166
169	240	149
160	271	165
171	185	166
165	203	162
148	202	169

145	225	171
154	242	165
168	163	166
163	168	160
164	168	157
161	157	165
156	180	180
171	145	182
160	166	168
161	169	180
156	191	178
163	174	165
143	152	185
157	165	172
164	147	172
166	138	162
160	197	163
151	145	160
154	165	171
160	177	165
167	192	160
147	142	175
154	132	165
152	138	168
144	151	168
164	157	169
154	134	172
176	177	169
208	166	166
231	172	157
223	214	158
226	172	160
142	168	168
174	191	172
249	189	160
229	212	152
192	234	154
167	177	172
169	185	172
163	203	165
199	233	166
212	275	166
217	205	171
161	291	166
160	288	166
166	217	163
154	195	143
150	203	177
175	252	169
166	305	157

155	292	157
149	311	163
162	211	174
177	285	168
171	297	172
169	305	172
151	260	165
148	291	168
178	302	159
152	283	177
145	217	162
163	200	157
174	263	168
172	249	166
172	225	158
175	263	146
168	257	165
174	275	166
174	268	168
174	237	151
162	237	148
151	248	162
163	189	169
165	256	160
172	286	154
169	237	157
166	183	157
155	265	166
172	322	160
162	271	163
160	103	173
162	280	168
162	86	174
160	155	162
168	172	163
163	165	148
168	169	169
162	166	159
188	177	158
188	166	166
179	171	154
169	174	172
179	172	177
188	168	156
171	174	151
185	168	171
172	179	154
180	178	163
177	174	160
162	165	166

162	165	160
168	168	162
156	172	172
180	182	168
151	169	163
178	160	169
175	178	175
163	172	165
160	171	162
168	172	168
169	169	172
177	171	162
163	174	166
151	177	160
157	165	157
152	174	171
139	168	163
143	169	166
156	178	160
158	174	160
176	169	159
160	171	165
157	172	180
171	183	177
179	166	180
163	165	154
152	169	162
160	171	160
152	168	160
160	160	175
155	166	165
158	162	157
162	168	154
162	176	158
169	160	157
162	162	174
166	162	160
174	172	165
151	171	163
162	166	154
165	172	165
162	169	160
158	172	163
142	169	155
145	169	140
145	163	154
145	169	169
148	180	155
154	166	165
149	174	156

152	177	152
145	183	149
148	168	165
129	176	169
145	162	183
150	166	178
146	169	166
148	172	183
140	168	177
137	163	188
148	157	183
131	163	160
137	169	166
138	160	180
131	158	178
148	163	174
145	152	172
135	162	188
157	157	172
145	157	169
151	152	180
140	154	178
140	154	186
150	157	182
149		182
134	154	174
134	154	165
152	154	178
145	163	192
158	158	169
142	157	178
143	158	176
138	154	186
135	157	191
138	157	169
142	160	178
148	157	187
146	149	166
135	157	185
146	162	188
146	154	172
148	154	185
145	156	166
140	163	175
142	158	182
151	152	191
145	160	163
149	152	180
138	157	188
142	155	183

142	158	165
140	162	165
142	160	146
131	157	183
137	160	165
138	171	160
140	175	152
139	188	176
134	166	169
142	163	162
139	177	169
134	172	172
143	151	175
143	194	172
146	200	169
139	163	155
142	177	149
136	177	169
142	171	169
138	174	163
145	174	174
151	178	171
140	169	162
154	171	163
160	182	174
160	166	169
138	188	160
151	177	179
138	154	187
152	163	160
145	188	166
151	188	145
151	191	177
138	157	179
143	200	177
152	180	182
143	189	154
131	185	174
148	188	152
151	149	160
155	186	160
154	191	172
153	183	179
134	166	158
149	155	162
135	205	169
143	188	179
143	194	175
135	185	169
	154	166

202	174
189	174
180	169
172	160
186	151
185	162
191	163
188	163
178	165
169	170
183	151
170	169
185	202
192	183
182	159
163	156
166	165
172	186
174	192
183	168
174	165
182	158
168	200
183	172
172	163
187	162
177	154
167	200
157	182
177	165
175	157
175	149
157	195
151	192
175	171
176	163
174	151
174	192
179	189
169	175
177	148
169	143
168	188
174	162
168	158
170	146
156	175
172	177
177	169
177	152

174	197
184	154
182	189
183	160
182	174
172	158
178	163
160	182
172	182
188	174
168	171
156	172
163	175
166	177
172	163
167	160
188	182
176	171
171	168
168	185
155	171
161	179
164	174
159	174
160	158
165	160
160	171
160	172
173	165
169	155
170	178
174	157
162	163
168	185
180	163
196	163
185	174
169	160
171	171
169	183
196	158
168	169
155	169
158	175
197	163
203	165
175	166
154	177
168	157
171	157

186	154
171	154
160	176
160	160
192	166
180	162
180	174
153	174
155	178
177	175
176	163
170	188
149	177
151	178
157	160
169	169
171	189
188	168
152	186
155	177
195	166
180	178
160	163
157	192
160	177
177	157
183	166
162	192
174	186
179	174
166	168
173	157
160	200
166	189
157	169
179	151
172	157
182	177
194	171
212	158
171	166
167	160
174	171
192	189
173	194
180	175
166	165
174	168
186	180
168	175

176	175
169	166
163	165
168	172
203	178
170	174
169	166
188	185
198	185
183	185
195	174
178	182
162	185
192	171
149	182
199	183
163	176
178	182
172	182
168	169
183	182
182	191
175	186
165	174
157	185
169	182
175	203
188	186
163	188
171	195
154	175
152	185
157	203
154	189
162	211
148	222
153	223
145	209
154	214
158	225
157	229
143	192
152	225
155	203
163	196
154	191
154	182
157	175
154	183
162	179

152	163
149	166
148	182
172	185
152	172
169	
175	
163	
169	
183	
175	
175	
165	
175	
175	
160	
162	
169	
180	
157	
158	
166	
171	
165	
162	
162	
163	
185	
186	
175	
163	
172	
180	
175	
165	
143	
182	
183	
180	
163	
166	
160	
179	
154	
163	
178	
178	
172	
155	
174	

175
180
157
174
177
188
191
160
165
180
183
186
182
185
192
186
177
174
174
182
183
191
183
185
160
165
179
169
180
154
163
172
166
172
163
157
182
172
177
155
159
186
172
160
157
178
157
173
176
152

175
168
189
194
182
178
189
179
191
188
183
179
192
191
171
172
182
163
142
158
176
183
142
145
157
177
174
146
162
176
185
183
154
155
148
152
162
149
157
168
157
149
165
171
166
158
154
166
176
172

160
162
159
172
171
163
165
169
166
166
180
154
166
163
174
163
157

Epidermal thickness [µm], day 4, f19mm

0 Gray	2 Gray	10 Gray
166	154	146
165	146	155
173	182	160
155	174	134
157	165	151
162	154	166
182	149	189
165	145	142
161	166	170
168	182	186
163	155	134
162	149	146
161	148	134
148	178	160
142	181	151
157	194	158
168	163	145
155	145	155
152	155	151
135	154	155
158	191	155
175	205	151
177	178	138
158	148	151
161	145	154
169	211	157
175	169	155
171	185	162
155	148	140
175	152	121
183	205	157
175	178	157
170	189	152
166	145	152
160	155	152
162	206	169
164	155	146
163	191	138
157	152	148
168	154	144
184	157	162
178	202	138
163	186	128
177	216	132
172	165	146
154	140	140
170	206	130
178	185	141

169	145	155
162	209	121
171	186	125
161	157	129
181	149	137
182	140	132
178	215	142
175	192	166
182	172	145
161	146	127
168	149	148
166	215	134
185	191	159
174	152	166
166	151	148
156	160	129
148	207	138
177	188	169
168	152	160
162	154	123
174	162	140
153	177	166
173	174	169
180	152	149
159	149	163
154	211	142
159	192	122
177	204	160
181	142	146
170	137	157
158	194	155
166	205	155
177	154	137
169	149	160
178	149	160
165	205	158
169	180	134
188	175	160
175	137	151
168	152	132
177	186	140
160	160	131
152	142	151
152	145	165
148	228	172
149	188	154
155	155	168
159	154	158
154	152	179
135	202	156

148	175	148
145	157	171
155	152	163
142	155	167
175	215	162
148	178	153
165	150	173
186	149	162
182	143	158
199	216	158
172	159	172
155	169	169
162	145	162
164	149	152
158	206	154
154	175	169
148	149	148
158	145	157
172	143	151
157	202	162
165	182	168
162	143	152
161	152	164
178	149	162
151	209	157
169	171	162
160	146	131
162	151	140
186	154	155
152	199	172
160	152	165
155	149	171
172	149	168
169	152	158
166	211	145
145	195	173
154	149	172
167	145	173
163	142	161
171	208	146
146	175	161
155	145	177
172	154	157
182	151	158
151	192	152
154	163	163
162	149	165
175	154	157
168	210	142
148	182	128

154	166	165
158	147	262
157	145	202
162	198	142
154	175	138
160	145	300
148	142	159
169	145	141
171	213	146
154	174	154
154	175	156
146	148	148
169	149	146
155	205	137
175	209	146
143	178	155
155	145	166
178	146	137
155	209	167
151	174	134
154	157	119
143	152	171
167	148	155
162	172	131
154	145	125
162	145	173
148	195	132
162	218	151
155	178	171
166	145	163
152	146	125
145	212	138
160	214	151
152	185	166
154	151	163
151	142	154
160	135	169
152	205	162
151	195	152
142	160	149
138	128	148
135	152	165
162	202	162
140	205	155
148	169	177
137	152	165
137	138	165
160	215	151
145	197	135
160	143	138

145	138	165
131	155	168
166	160	160
151	148	132
158	148	135
158	202	154
140	223	169
169	215	158
162	185	169
167	174	165
140	152	192
140	151	138
158	208	123
152	189	145
161	175	160
142	162	166
158	183	155
182	205	146
155	186	172
187	185	157
164	174	152
178	168	154
157	206	154
157	194	146
164	177	152
143	168	158
152	202	175
145	200	160
153	195	161
163	169	150
140	192	162
158	202	149
157	195	150
145	195	158
165	163	148
145	175	153
142	209	148
143	175	163
143	168	186
163	166	147
143	160	152
142	202	151
151	175	154
158	168	154
166	160	152
142	154	165
145	202	157
157	194	166
143	182	157
168	185	142

146	152	160
146	172	153
155	177	152
140	158	135
171	208	148
163	172	140
157	166	143
143	166	157
163	163	161
171	195	145
162	212	142
145	162	152
152	174	132
158	163	142
171	192	142
146	182	145
161	171	142
154	174	146
172	175	134
158	192	126
158	162	129
157	165	118
176	206	138
184	186	138
167	192	142
180	166	131
179	172	128
148	206	135
157	188	145
151	163	126
137	168	142
132	142	126
152	203	137
151	177	122
155	188	89
155	169	111
146	162	126
158	205	154
155	182	154
162	160	155
137	186	158
145	208	157
158	191	145
157	180	154
154	180	148
151	172	137
148	189	123
157	202	128
145	158	146
158	174	137

145	155	140
146	214	129
154	174	137
146	183	140
171	163	138
151	178	129
149	208	126
155	189	143
146	171	146
168	185	137
163	165	140
149	177	137
148	168	122
157	186	120
143	188	134
154	205	227
138	175	230
155	168	132
149	197	123
146	205	123
148	189	217
158	188	235
185	171	160
169	195	171
164	218	155
187	186	148
184	172	152
148	174	235
149	189	195
160	205	273
157	189	165
148	172	190
162	209	174
151	192	190
146	211	265
163	198	176
157	175	278
146	194	165
140	180	188
172	211	275
160	183	187
155	165	148
165	208	145
149	205	160
145	202	169
151	178	142
163	177	129
154	185	185
137	211	253
149	178	172

148	169	164
135	198	135
186	171	165
170	203	195
178	189	149
173	177	260
177	166	195
168	174	182
211	200	178
171	182	257
191	174	129
162	186	278
185	166	186
172	162	154
185	177	179
188	194	158
178	169	252
155	157	163
176	211	186
178	166	246
161	149	138
192	154	172
186	183	228
186	185	270
176	169	138
165	169	174
192	152	151
191	183	158
199	183	174
178	188	256
178	146	157
201	158	245
154	162	182
198	152	155
190	160	195
179	149	169
169	160	245
167	163	176
197	152	195
181	152	168
182	152	166
169	152	240
186	177	155
193	152	198
180	162	251
170	166	175
153	178	197
169	146	165
182	151	175
181	160	262

153	152	203
181	155	163
180	162	195
195	157	150
184	157	183
164	152	265
162	157	194
195	163	159
194	160	182
201	175	248
181	154	272
174	158	190
185	168	202
194	155	263
194	146	198
180	165	152
183	157	202
169	163	159
218	194	191
201	162	267
202	200	266
180	158	203
167	162	166
192	212	185
197	185	263
185	175	277
202	143	174
152	145	157
215	163	186
203	165	250
192	160	265
182	165	166
155	169	204
210	180	155
191	185	180
182	157	257
158	146	188
192	200	166
198	188	168
187	194	182
180	171	175
178	134	202
189	151	175
195	163	182
196	142	180
178	154	192
182	142	228
172	149	178
187	143	142
186	142	183

180	155	172
193	145	214
175	151	251
180	182	273
188	186	287
177	315	172
174	296	196
160	192	239
198	188	268
185	175	262
178	197	180
182	294	172
179	320	206
188	178	152
179	160	149
192	166	175
178	317	158
187	246	155
166	151	155
178	158	158
172	148	161
163	148	166
165	149	167
175	175	160
166	171	161
166	182	169
165	314	166
192	291	158
171	175	156
170	171	169
178	172	152
185	242	171
179	163	165
169	182	160
182	160	169
158	154	168
168	142	157
161	131	168
188	125	168
214	157	166
176	154	162
174	163	163
201	180	163
183	157	151
175	166	165
181	146	167
178	152	168
183	138	171
179	174	165
168	154	158

163	143	160
174	169	162
175	157	160
175	145	162
183	131	165
177	142	169
195	126	160
176	131	157
158	154	166
178	134	158
177	158	166
203	177	160
172	157	157
169	142	165
168	142	166
165	163	160
182	168	163
186	160	165
175	137	168
167	132	162
181	132	166
174	143	168
173	149	162
179	168	169
170	169	178
179	155	163
196	148	155
178	140	162
171	149	162
168	143	171
175	165	151
188	137	163
169	160	163
174	149	166
178	269	173
169	152	154
153	169	162
181	171	153
159	165	136
185	169	175
177	165	162
153	165	168
177	151	157
182	152	147
195	157	171
178	168	172
151	165	152
205	151	146
204	163	135
198	157	162

182	152	171
205	168	152
155	154	145
189	155	131
198	155	172
177	158	166
178	162	151
202	165	147
198	162	131
188	161	162
168	169	145
162	179	145
176	164	128
192	162	158
188	169	156
167	175	155
163	151	145
187	145	132
188	164	156
178	162	165
168	177	145
171	146	150
156	166	139
184	170	153
191	146	152
169	179	166
168	158	143
179	156	140
190	177	134
169	175	144
162	184	176
174	174	125
160	162	169
203	186	111
180	152	144
162	163	172
170	148	122
194	151	143
186	148	128
158	143	157
201	149	155
176	145	142
167	148	126
186	171	117
172	160	135
202	162	148
199	157	151
174	146	143
180	158	163
162	175	115

172	160	106
183	169	142
179	142	131
177	162	147
172	165	146
167	169	122
188	166	112
177	143	137
205	158	172
183	149	131
167	155	110
158	148	110
168	165	117
228	163	153
173	145	117
182	152	118
172	132	154
225	151	162
234	138	140
179	155	118
188	138	148
169	122	142
171	135	167
214	166	157
188	144	131
186	140	143
182	146	183
158	149	152
241	137	154
187	162	155
177	155	141
188	142	172
208	143	151
218	164	162
201	153	155
179	138	158
169	155	169
192	155	158
225	135	158
188	149	172
189	145	152
177	152	160
215	142	171
183	175	162
234	175	165
193	166	169
190	136	166
178	174	166
213	172	166
193	158	169

166	162	162
192	165	171
202	158	166
227	179	166
199	143	163
185	178	172
172	178	171
188	146	168
238	173	158
180	145	171
171	129	165
173	152	166
204	162	166
232	157	169
220	151	197
187	151	171
187	152	165
178	135	160
204		163
219		163
177		165
184		155
185		168
192		160
220		155
202		172
175		163
177		168
180		169
220		174
186		154
185		162
178		166
203		166
192		146
180		162
191		168
164		158
219		158
184		154
162		166
153		168
171		163
209		163
179		165
195		162
153		182
168		160
202		174
175		186

176	152
184	178
213	171
227	143
183	162
178	162
170	183
231	162
188	166
181	175
228	154
212	183
185	164
180	169
177	169
174	172
168	178
177	160
184	188
185	154
178	174
193	189
183	154
183	155
168	169
162	166
153	165
174	172
173	172
172	158
159	186
170	168
174	169
182	166
185	151
169	154
186	165
177	148
173	148
172	138
178	142
176	148
171	165
	163
	171
	149
	129
	149
	134
	135

168
151
148
154
154
183
135
143
151
154
162
168
175
172
157
185
163
175
177
182
186
174
171
180
172
165
151
166
166
165
160
165
162
155
177
188
172
163
154
163
165
171
191
155
185
177
155
177
169
166

158
160
129
125
146
122
140
151
149
171
178
174
158
152
158
162
162
168
157
160
160
162
157
169
171
152
163
163
163
158
152
134
171
162
145
160
148
160
165
158
148
160
167
157
148
155
161
139
152
142

131
132
142
155
148
155
142
148
192
154
157
151
148
142
151
152
167
152
155
145
157
160
158
148
151
142
151
149
155
142
168
155
146
132
148
152
162
160
142
132
156
139
172
138
148
162
158
160
146
145

139
162
175
155
154
135
155
162
164
142
145
157
149
165
151
183
140
163
169
174
135
158
175
163
174
182
181
154
155
180
177
163
165
160
162
151
172
163
151
169
143
167
167
166
160
150
167
182
169
173

151
155
164
163
168
163
162
166
170
166
163
150
173
148
149
149
150
168
180
169
148
187
182
175
180
169
174
163
169
163
166
200
148
158
155
175
194
157
166
149
140
159
154
165
154
143
151
152
154
138

145
160
160
154
151
163
160
145
149
162
169
166
158
162
175
169
180
175
163
188
166
165
158
148
162
163
160
151
166
163
146
171
173
172
169
169
169
148
154
166
160
166
154
158
146
169
169
160
155
163

162
175
165
158
138
157
157
169
172
172
165
160
172
157
180
151
145
166
169
155
177
168
160
169
168
146
165
160
154
169
148
160
160
160
154
163
166
160
149
152
171
171
154

Cornified Layer thickness [µm], day 2, f19

0 Gray	*2 Gray*	*10 Gray*
55	29	43
29	34	49
32	34	38
27	25	49
20	37	51
16	38	49
21	28	46
22	40	36
34	45	42
33	26	
40	34	
	34	
	25	
	28	
	23	
	34	
	29	
	35	
	42	

Cornified layer thickness [µm], d4, f19mm

0 Gy	*2 Gy*	*10 Gy*
42	69	46
77	62	43
65	62	35
68	54	32
60	62	48
68	65	34
68	49	51
55	66	26
68	65	34
56	59	19
56	63	20
51	52	16
45	65	23
52	65	29
40	58	40
47	52	28
46	57	34
53	49	40
43	46	28
34	37	57
45	45	51
50	35	49
29	38	32
32	60	31
37	31	65
28	66	51
32	48	58
23	60	54
34	62	59
35	78	55
31	52	55
35		57
51		45
32		49
31		62
38		48
31		43
51		48
31		54
34		46
43		52
48		51
40		43
48		23
43		42
46		29
39		17
38		52

45	45
40	52
37	54
51	62
49	54
34	57
50	58
32	55
40	49
46	52
37	54
43	48
40	69
45	46
40	52
43	55
40	60
77	52
40	45
38	34
43	62
31	55
38	51
49	62
43	57
71	32
65	31
74	40
71	23
65	43
70	35
65	26
71	42
76	29
59	69
65	58
70	42
56	49
60	45
60	54
59	52
60	51
65	55
70	65
65	42
70	66
54	54
59	39
63	37
48	40

62	53
58	40
65	43
49	49
68	54
60	54
47	65
62	55
42	37
60	31
53	43
56	32
56	45
53	51
71	59
57	59
55	45
71	43
70	59
77	60
88	40
86	18
81	45
89	58
90	46
82	51
109	51
115	55
78	44
103	56
81	37
101	23
94	34
88	25
81	31
93	26
102	34
87	28
87	29
84	37
96	37
103	43
111	32
79	52
81	40
76	45
76	45
71	46
81	51
	54

52
46
59
51
34
62
49
43
60
40
45
22
46
55
57
52
54
52
55
49
51
52
52
54
46
57
46
57
48
43
40
